# Using coevolution to improve protein subfamily classification

**DOI:** 10.1186/1471-2105-16-S8-A6

**Published:** 2015-04-30

**Authors:** Franco Simonetti, Martin Banchero, Ariel J Berenstein, Ariel Chernomoretz, Cristina Marino Buslje

**Affiliations:** 1 Fundación Instituto Leloir, Buenos Aires, Argentina; 2 Universidad de Buenos Aires, Buenos Aires, Argentina

## Background

The common approach for protein subfamily classification relies on grouping protein sequences according to their degree of similarity. However, there is no single sequence similarity threshold for accurately grouping sequences into isofunctional groups.

Current subfamily classification methods use bottom-up clustering to construct a cluster hierarchy, then cut the hierarchy at the most appropriate locations to obtain a single partitioning. These methods usually integrate data such as protein sequence similarity, residue conservation within groups and HMM profiles. Despite this straightforward approach, results usually predict a great number of subfamilies with few members and limited biological meaning.

The goal of this study is to identify subsets of functionally related sequences within a given superfamily. Since all proteins within a superfamily share a common ancestor, we hypothesize that functional diversity within superfamilies has arisen through a series of concerted changes that must have left an identifiable coevolutionary signal.

## Material and methods

The challenge is to be able to separate the subfamilies coevolutionary signals and use them in the process of subfamily classification. This information can be used to guide a hierarchical clustering. Our approach uses Mutual Information to calculate covariation and commonly used clustering methods based on sequence similarity. We have defined a select group of superfamilies from the Structure Function Linkage Database as our gold standard dataset.

## Results

Different approaches were considered for integrating Mutual Information data in sequence clustering. Since Mutual Information can only be calculated for a group of sequences, a preliminary sequence clustering is performed. Using solely covariation data, our method can cluster groups of sequences from the same subfamily. For a complete clustering solution, it performs almost as good as a hierarchical clustering based on sequence similarity. The next step will be to integrate both methods.

## Conclusions

Automated protein classification remains an active topic of research and state of the art methods are far from predicting biologically meaningful results. Covariation data has never been used before in this context and further analysis are needed to improve the method.

